# Topics of Public Interest

**Published:** 1916-01

**Authors:** 


### TOPICS OF PUBLIC INTEREST.

**County Tuberculosis Hospitals.** From information supplied by the State Charities Aid Assn., we learn that recent additions are as follows: Jefferson Co., $25,000 appropriated supplemental to previous appropriation of $15,000, site purchased, hospital to be built within a few months. Rensselaer Co., $150,000 appropriated for 120 bed hospital, site selected, construction will probably begin in spring. Present institution on alms house farm filled to capacity, not satisfactory, and usually has a waiting list. Chenango Co., $20,000 bond issue authorized, site selected. Niagara Co., $100,000 voted. Rockland Co., $50,000 voted. Steuben Co., $30,000 voted. Herkimer Co., $25,000 voted. Chautauqua Co. has accepted a gift of $150,000 for a tuberculosis hospital. Outside of New York City, 32 counties now have tuberculosis hospitals in operation or assured for the near future. The 25 remaining counties have rather sparse populations and most of them are so hilly or mountainous that proper accommodations for tuberculous patients could easily be improvised.

**Diminution in Mortality.** The Bureau of the Census states that the death rate for 1914 in the Registration Area was 13.6: 1000 population, the lowest recorded. The average, 1901-05 was 16.2 and for 1906-10, 15.1. Still more favorable results would be shown if the Registration had remained as it was.

**Incomes.** A slight diminution occurred in the number of persons paying income taxes in 1914 as compared with 1913: 357,515 as compared with 357,598, but the total amount paid was $16,559,000 as compared with $12,728,000. It is perhaps unfortunate that incomes above 500,000 paid $6,439,000 instead of $3,437,000, on the ground that increase of wealth already enormous, is rather detrimental to the general prosperity. However, as the tax for 1913 began with March 1 and as perhaps the majority of dividends are payable January 1 and July 1, this may account for the apparently abnormal rise. 174 incomes of $500,000 or more were reported. It will be noted that only about 3½ per mille of the population or about 1½% of the families of the country, have incomes of $4000 or more. Probably the majority of this 1½% think of themselves as poor, and with considerable excuse.

**N. Y. State Medical Library.** Since the fire in 1911, 19,600 bound volumes, several thousand pamphlets and reprints and much unbound material has been accumulated. About 500 periodicals are received and more than 200 have complete files. Thus, the library has more than made good its loss. A printed list of complete sets of journals has been issued. Books are sent by mail on request, to licensed physicians, full-time instructors in medical colleges, internes, registered and certified nurses and those engaged in research who present suitable references. The only expense is for return postage. Books may be retained 4 weeks, periodicals 2 weeks. Physicians coming from a distance to consult the library may have material reserved in advance. Gifts of books, periodicals and reprints are solicited. Address for all these purposes: Medical Librian, State Library, Education Bdg., Albany.

---

**U. S. Public Health Service: Stereopticon Loan Library.** Any responsible person may borrow standard slides, 3¼x4 inches, or photographs from which slides may be made. Catalogue will be sent on request. Address Surgeon General, U. S. P. H. S., Washington, D. C., referring to letters "D. Q." The following subjects are available: Alaska, 83 views; Children and Children's Diseases, 50; Health Exhibits, 90 duplicating the exhibit at the Panama-Pacific Exposition; Hookworm, 90; Indians (housing and living conditions), 50; Leprosy, 45; Malaria, 275; Milk, 80; Parasites and Organisms, 200; Pellagra, 60; Plague, 500; Activities of the U. S. P. H. S., 320; Small Pox, 90; Trachoma, 120; Tropical Diseases, 40; Tuberculosis, 100; Typhoid, 350; Yellow Fever (number not stated). Various miscellaneous slides are also available and selections may be made from the various series so as to illustrate other subjects. The Service will assist in the selection of slides.

---

**Football** caused 16 deaths in the fall of 1915, mostly in minor games and among preparatory school and miscellaneous teams. It has been estimated that the few games of the larger colleges cost, on an approximate average $50,000 per college. For the few prominent football universities, the cost is considerably more. This direct cost is insignificant in comparison with that for transportation and accommodation, bets, etc. Some extremists have suggested that the total cost might better be applied to military preparedness. With reference solely to mortality and traumatic disability, we question whether the game is worth the shot. The actual danger of football could be almost absolutely prevented by making football a game of direct muscular skill instead of inhibition. Solely from the standpoint of sport, we would also favor the idea of putting football on the same basis as baseball, quite aside from the question of safety to the players. This point can be made clearer by a negative illustration. Imagine the pitcher elaborately poised to throw a curve. At the crucial moment, half a dozen of the opposing team precipitate themselves upon him.

Granted that, occasionally, the ball is actually pitched an extra member of the in team bats the bat of the batter, occasionally fracturing his skull by mistake. Granted that the batter manages to hit the ball or otherwise by some extension of the rules of strikes and balls to be due at first base, he runs through a mob of infielders seeking to impede his progress. In the outfield, the picturesque catch of a fly is prevented by a skillful kick on the elbow . . . and so on for the various stages of the game. We doubt if many attendants on baseball games would favor such a change. Now imagine that football were recognized on the same principle as baseball, that every detail of the game were a matter of individual skill and that no deliberate impeding of the individual play was allowable. Under such circumstances, football would become a spectacular performance which would appeal even to the inexperienced observer, it probably could be played for a longer season and in decent weather and it would lose its lethal and most of its surgical aspects. However, there is no gain without some loss and oratory would lose a number of metaphors such as team-work and hitting the line hard.

---

**U. S. Public Health Service Examinations** for assistant surgeon, will be held January 24, at Washington and various other places, to be designated according to applications received. Candidates should address the Surgeon General at Washington, immediately. The salary is $2000, increased by promotion, the tenure being permanent.

---

**Horse Flesh** will be allowed to be sold in New York State, after January 1, 1916, but only under its own name. This is a sensible action as it will tend to increase the amount of cheap and good meat. The horse is not liable to tuberculosis or other infection except tetanus and certain very rare and easily diagnosticable diseases so that it is, on the whole, one of the safest meats. The prejudice against horse meat is purely sentimental and largely owing to the personal feeling toward the horse, now decadent since the general introduction of horseless vehicles. As the horse cannot be profitably raised for meat, slaughter is practically limited to crippled and superannuated animals so that the meat is usually rather tough and tasteless. It contains more glycogen than most meat on the market. It is particularly adapted to making dried, smoked, "beef."

---

**The Factor of Poverty in Sanitation** was discussed before the 24th annual meeting of the Clinical Society of Surgeons in Washington, November 24, by Surgeon General Wm. C. Gorgas. He alluded to the removal of competition by epidemics and war and predicted a general rise of wages in Europe following the present war. He quoted a cabinet officer to the effect that 55% of the arable land of this country was kept out of use and advocated a tax on land that would put it into use. (Note: Without discussing the single tax or the matter of wages, it may be pointed out that the average skilled laborer works fewer hours, with less exertion and anxiety, and has more pleasures than the average small farmer of 50-100 acres; that the average unskilled laborer is, on the whole, better off than the hired man. In order to induce laborers in the city to make a general move toward the country that would be successful, it would be necessary first to have a confiscatory single tax, secondly to remit the tax after the land was occupied, thirdly, to furnish buildings and implements, fourthly, to teach the laborer a new trade).

---

**Two Million Automobiles** were being operated in the U. S., July 1, 1914.

---

**Preparedness.** This country needs a larger military force, solely to insure against invasion. Either we do not need any increase at all or we need a very large one. A country that is absolutely defenseless and that appeals for peace purely as a matter of ethics, is more apt to be considered sincere than one that speaks softly but carries a big stick. If invaded, such a country is more apt to receive considerate treatment than one that has put up an ineffectual fight. Granted that we do need a defensive force, the regular army should theoretically be increased up to the requirements of "first line" defense. As to the second line, we feel strongly that the National Guard and not a new organization should be utilized. The National Guard, originally springing up as a spontaneous recognition of the need of a trained and not merely a paper militia, has gradually developed, first into an available state force, and then into one of national value. The men who have labored for years to render it efficient, deserve first consideration. No one recognizes more clearly than themselves that it should be enlarged and made more efficient. It is a matter of common sense to use existing forces so far as possible, in any enterprise. A strong "second line" of national defense could be developed more quickly and more economically by developing the National Guard than by starting fresh. The training of the medical profession for military service is an important item, and it can be better done by the assistance of the National Guard than in an organization without precedents and with no training at all. We invite further discussion of this important subject.

**Decrease of Tuberculosis in Ontario.** The rate of deaths per 100,000 population has diminished from 148 to 85 in the last 11 years.

---

**Christian Science and Surgery.** One of the strangest aftermaths of the Haiselden case is that the Christian Science Monitor condemns the failure to operate to relieve the congenital intestinal occlusion.

---

**Expulsion on Ethical Grounds.** Dr. H. J. Haiselden, who advised against operating on the defective Bollenger baby in Chicago, has been expelled by the Chicago Medical Society, on account of the publicity methods employed, not as a protest against his conduct of the case. We were disgusted—and interested—to see Dr. Haiselden in moving pictures and intended to make a few scathing remarks until we reflected that our own picture and that of many of our most highly esteemed colleagues have appeared in newspapers, and that there is no essential ethical difference in this respect. While we feel that the degree of publicity given to this case was unwarranted, we also feel that ethics ought to be subject to definite rules of principle, rather than of degree and that these rules should apply equally to all members of our profession.

---

**Philippine Civil Service Examinations.** The positions of Bacteriologist and Pathologist, salary $2,000 and $2,500, and of Assistant Surgeon, $1,800, are open. No formal examination will be held but applicants will be rated according to education, experience and publication of thesis submitted. Apply immediately to the U. S. Civil Service Commission, Washington, for form B. I. A. 2 and 2118.

---

**Kidney Cures Seized** by U. S. Dept. of Agriculture. "Old Jim Field's Phosphate, Dil and Gin, Mankind's Greatest Friend," and Stuart's Buchu and Juniper Compound, have been condemned under the Pure Food and Drugs Act, the former containing 41%, the latter 18% of alcohol. Such proportions of alcohol were held to be positively harmful.

